# Prediabetes and diabetes prevalence and risk factors comparison between ethnic groups in the United Arab Emirates

**DOI:** 10.1038/s41598-019-53505-7

**Published:** 2019-11-25

**Authors:** Rifat Hamoudi, Narjes Saheb Sharif-Askari, Fatemeh Saheb Sharif-Askari, Salah Abusnana, Hayat Aljaibeji, Jalal Taneera, Nabil Sulaiman

**Affiliations:** 1 Sharjah Institute of Medical Research, College of Medicine, University of Sharjah, Sharjah, United Arab Emirates; 2 College of Medicine, University of Sharjah, Sharjah, United Arab Emirates; 3 Baker Heart and Diabetes Institute, 75 Commercial Road, Melbourne, Victoria 3004 Australia

**Keywords:** Metabolic disorders, Epidemiology, Risk factors

## Abstract

The economic growth has paralleled the rise of diabetes and its complications in multiethnic population of United Arab Emirates (UAE). Previous studies have shown that characteristics of diabetes is variable across different ethnicities. The objective of this study was to compare diabetes prevalence and risk factors between UAE nationals and different expatriate’s ethnic groups in UAE using data from UAE National Diabetes and Lifestyle Study (UAEDIAB). The UAE nationals made one-fourth (n = 797, 25%) of total cohort and the remaining 75% belonged to immigrants. Across different ethnicities, adjusted prevalence of prediabetes ranged from 8% to 17%, while adjusted prevalence of newly diagnosed diabetes ranged from 3% to 13%. UAE nationals, Arabs non-nationals and Asians had the highest number of pre-diabetic as well as newly diagnosed diabetic patients. Adjusted prevalence of diabetes was highest in UAE nationals (male 21% and female 23%) as well as Asian non-Arabs (male 23% and female 20%), where 40% of both groups fell under the range of either prediabetes or diabetes conditions. Multivariate factors of diabetes versus non-diabetes included older age, ethnicities of Asian non-Arabs and local UAE nationals, family history of diabetes, obesity, snoring, decreased level of high density lipoprotein, elevated levels of triglycerides and blood pressure. In conclusion, diabetes prevalence and risk factors vary across the different ethnic groups in UAE, and hence interventions towards identification and prevention of diabetes should not treat all patients alike.

## Introduction

The burden of diabetes is rising globally, most markedly in developing countries that are undergoing industrialization and have shifted to more sedentary lifestyle, poorer eating habits as well as less physical activity. The global number of adults with diabetes increased from 108 million in 1980 to 422 million in 2014^1^. According to International Diabetes Federation (IDF), currently 425 million people have diabetes in the world and more than 39 million people in the Middle East and North Africa region (MENA) Region^2^. United Arab Emirates, one of the 19 countries of the IDF MENA region, has experienced rapid economic growth and infrastructure expansion, following the discovery of oil^3^. This economic burst parallels the rise of diabetes and its complications in UAE with diabetes numbers reaching over 1,185,500 cases in 2017^2^.

The UAE population is made up around 20% local UAE nationals and the reminder non-UAE immigrants from different Arabs and non-Arabs (Asians and Westerners) ethnic backgrounds^4,5^. Previous studies have shown that prevalence as well as risk factors of diabetes is variable across different ethnicities^6,7^. Although a number of previous studies have attempted to describe diabetes in UAE^8–10^, these studies mainly focused on UAE nationals and few have included non-nationals in to perspective^11^. The UAE National Diabetes and Lifestyle Study (UAEDIAB) study was conducted in two phases; the first phase was conducted among immigrants non-UAE population and the second phase was conducted among population of UAE nationals^12,13^. For purpose of this study we merged the data from phase one and two.

The objective of this study was to compare the prevalence and risk factors of diabetes between UAE nationals and different expatriate’s ethnicities living across United Arab Emirates using data from the UAEDIAB.

## Methods

### Study design, population and settings

The UAEDIAB was a cross-sectional study conducted to investigate the prevalence and risk factors of diabetes among UAE nationals and expatriates who have been living in Sharjah, Dubai, and Northern Emirates for at least four years. The UAE multi-ethnic expatriate population were surveyed in the first phase, and the UAE Emiratis were surveyed in the second phase. Ethical approval for this study was obtained from both University of Sharjah ethics committee on 23 June 2010, and UAE Ministry of Health on 14 March 2012, and all research in this study was performed in accordance with relevant guidelines and regulations. Before the interview, every participant read a detailed information sheet and signed an informed consent form to give information as well as blood samples^12^.

The details of study design and sampling of the UAEDIAB investigation are described elsewhere^12,14^. In the first phase, the target population was all non-UAE national adults aged 18 years and older residing in UAE applying for their second or subsequent visa renewal (i.e. resident for at least 4 years). The second phase of the study involving UAE nationals had a similar inclusion criteria’s but did consider the number of years of stay in the country. People with serious physical disabilities, learning disorders, severe communication barriers, and pregnant women were excluded from both phase one and two.

During a face-to-face interview, a designed questionnaire was used to collect sociodemographic data on gender, nationality (based on country of origin), date of birth, marital status, residence, family history of diabetes in first degree relatives and lifestyle habits. After completion of the interview, measurements of weight, height, waist and hip circumference and systolic and diastolic blood pressure were obtained and a fasting blood sample was collected to test levels for plasma glucose, HbA1c and lipids^12^.

### Variable definitions

Diabetes status was determined using measurements of FBG, HbA1c and participants self-report information. Following the WHO criteria for both of FBG and HbA1c, FBG < 6.1 mmol/l was considered normal, 6.1to 6.9 mmol/l was impaired fasting glucose (IFG), and ≥7.0 mmol/l indicated diabetes. Whereas, HbA1c, <6.5% was considered non-diabetic, and ≥6.5% diagnostic of indicated diabetes. Diabetes disease was classified into newly diagnosed diabetes mellitus (NDM) and known diabetes mellitus (KDM). Prevalence of total diabetes mellitus included both newly diagnosed and known cases of diabetes. KDM^15^ was assigned to those subjects who had previously been informed of having diabetes via a health professional and were either using diabetic medications or had a HbA1c ≥ 6.5% or FPG ≥ 7.0 mmol/l. Whereby participants who had FPG or HbA1c levels within the diabetes range and who had never been informed by a health professional that they had diabetes were classified as NDM following cuts off values provided by American Diabetes Association. For waist circumference different cut offs were used for Asians and the other ethnicities. For the waist circumference (WC) ethnic-specific cut offs were used where the high WC was defined as ≥90 cm in men and ≥80 cm in women from Asian ethnicity while the high WC for the rest of ethnic groups was defined as ≥102 cm in men and ≥88 cm in women. Body mass index (BMI) was obtained by dividing a participant’s weight in kilograms by the squared height in meters. To assess snoring and sleep apnoea, participants were asked whether they snore loudly or if anyone had observed them stop breathing during sleep. Hypertension was described either by a systolic blood pressure of >140 and or a diastolic blood pressure of >90. Triglycerides of >1.7 mmol/L was considered abnormal, while total cholesterol level >5.0 mmol/L was considered high. HDL < 1.0 mmol/l for males and <1.3 mmol/l for females was considered low, while LDL was categorized into three groups: <2.59, 2.59–3.34 and ≥3.34 mmol/l^12^.

### Statistical analysis

In this study, using the direct standardization method, the age standardised prevalence of prediabetes and diabetes (known and newly diagnosed diabetes) was calculated for UAE nationals as well as each of non-UAE national immigrant ethnic group using the world mid-year population of 2013^16^. Counts and percentages were used to present categorical variables, whereas normally distributed continuous data were presented as mean ± standard deviation (SD) and median and interquartile range (IQR) if their distribution was skewed.

In the univariate analysis, continuous data were analysed by Student t-test while the categorical data was compared using *χ*^2^test. Independent factors of DM were identified through development of a logistic regression model using enter method, which was adjusted for age, gender, and any other variables that in univariate analysis were significant at *P* < 0.05.The Statistical Package for Social Sciences (SPSS) version 24 (IBM Corp, New York) were used to carry out all the analysis.

## Results

**Figure 1:**
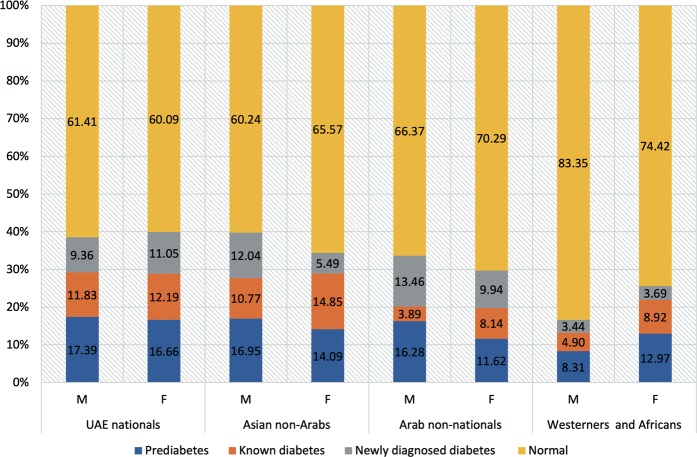
Flow chart of subject selection from UAE National Diabetes and Lifestyle Study. UAE, United Arab Emirates; UAEDIAB, UAE National Diabetes and Lifestyle Study.

The UAEDIAB study, which consisted of two phases, has screened over 3000 patients from two populations of national Emirati and expatriate living in different cities of UAE for risk of diabetes (Fig. 1).

After exclusion of cases with incomplete data, 313 from phase one and 187 from phase two, the total subject included were 2406 (75%) cases from phase one and 797 (25%) cases from phase two.

**Figure 2:**
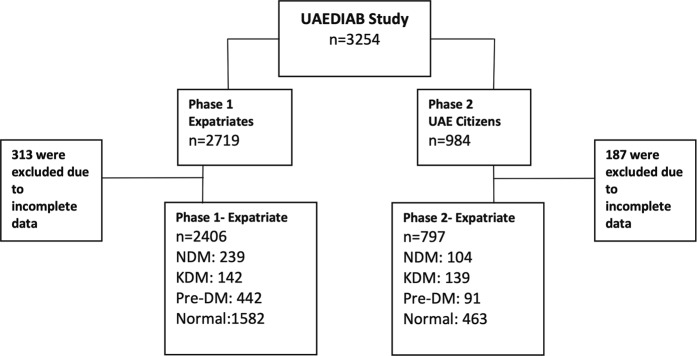
Age standardised prevalence of prediabetes and diabetes (known and new cases) across different ethnicities of local UAE nationals and immigrant non-UAE. UAE, United Arab Emirates.

The age standardised prevalence of prediabetes and diabetes (known and newly diagnosed diabetes) was calculated for UAE nationals as well as each of non-UAE national immigrant ethnic group (Fig. 2). Among UAE nationals, 42% (n = 334) were assessed to have either prediabetes or diabetes. The adjusted percentage of local female with diabetes was 23% out of which 11.5% were newly diagnosed cases while percentage of local males 21% out of which 9% were newly diagnosed cases.

The UAE nationals made the one-fourth (n = 797, 25%) of UAEDIAB total cohort and the remaining 75% (n = 2406) belonged to immigrants. The population of UAE expatriates was made up of the following four distinct ethnic groups: Arab non-nationals (n = 640, 20%), Asians non-Arabs (n = 1683, 52%), Westerners (n = 56, 2%) and Africans (n = 27, 1%). The total standardized prevalence of diabetes among immigrants was highest among Asians (male 23% and female 20%) followed by Arabs non-nationals (male 17% and female 18%). The fraction of population affected by either prediabetes or diabetes among Asians was nearby 40%, which was similar to UAE nationals. However, among Arab non-nationals and other ethnic minorities there was lower rate of prediabetes or diabetes.

In this study, we first compared characteristics of UAE national diabetics with diabetics from non-national ethnicities of Arabs non-nationals, Asians, African and westerners. Compared to UAE nationals, Arab non-nationals had higher numbers of males (54% versus 68%; *P* = 0.022), more active status (23% versus 63%; *P* < 0.001), higher total cholesterol (40% versus 66%; *P* < 0.001) but less snoring (95% versus 39%; *P* < 0.001). The next major immigrant ethnic group was the Asian non-Arabs, and compared to UAE nationals, Asians were on average 7 years younger: 51 ± 14 years vs 44 ± 11 years (*P* < 0.001), they were predominantly male (54% versus 87%; *P* < 0.001), reported lower family history for diabetes (64% versus 39%; *P* < 0.001), had lower BMI but larger waist circumference (63% versus 74%; *P* = 0.007), had higher levels of total cholesterol (40% versus 50%; *P* = 0.033) but lower levels of HDL (54% versus 43%; *P* = 0.014). The comparison for minor ethnic groups of Westerners and Africans are not reported here but they are displayed in the Table 1.

**Table 1:** Comparison of socio-demographic and clinical characteristics of diabetes between UAE national patients and immigrant ethnic groups of Arab non-nationals, Asian non-Arabs, Westerners and African.

Variable	DM Arab non-national n = 99	DM UAE locals n = 243	*P* value	DM Asians n = 272	DM UAE locals n = 243	*P* value	DM Westerners n = 5	DM UAE locals n = 243	*P* value	DM Africans n = 5	DM UAE locals n = 243	*P* value
Age (years) mean ± SD	49 ± 10	51 ± 14	0.184	44 ± 11	51 ± 14	<**0**.**001**	39 ± 16	51 ± 14	0.057	38 ± 13	51 ± 14	**0**.**035**
Male, n (%)	67 (68)	131 (54)	**0**.**022**	237 (87)	131 (54)	<**0**.**001**	2 (40)	131 (54)	0.665	3 (60)	131 (54)	0.787
Female, n (%)	32 (32)	112 (46)	**0**.**022**	35 (13)	112 (46)	<**0**.**001**	3 (60)	112 (46)	0.665	2 (40)	112 (46)	0.787
Family history of diabetes, n (%)	55 (56)	155 (64)	0.178	91 (33)	155 (64)	<**0**.**001**	0	155 (64)	**0**.**007**	3 (60)	155 (64)	0.862
Activity, n (%)	62 (63)	55 (23)	<**0**.**001**	136 (50)	55 (23)	<**0**.**001**	2 (40)	55 (23)	0.324	3 (60)	55 (23)	0.085
Snoring, n (%)	39 (39)	231 (95)	<**0**.**001**	78 (29)	231 (95)	<**0**.**001**	2 (40)	231 (95)	**0**.**002**	0	231 (95)	<**0**.**001**
BMI (kg/m^2^), n (%)			0.485			<**0**.**001**			0.977			0.367
Normal	13 (13)	43 (18)		87 (32)	43 (18)		1 (20)	43 (18)		2 (40)	43 (18)	
Overweight	31 (31)	80 (33)		117 (43)	80 (33)		2 (40)	80 (33)		0	80 (33)	
Obese	55 (56)	118 (49)		66 (24)	118 (49)		2 (40)	118 (49)		3 (60)	118 (49)	
Waist circumference high, n (%)	64 (65)	152 (63)	0.892	200 (74)	152 (63)	**0**.**007**	2 (40)	152 (63)	0.359	4 (80)	152 (63)	0.657
HDL, n (%)			0.074			**0**.**014**			0.801			0.664
<1 for males & < 1.3 for females	56 (57)	111 (46)		154 (57)	111 (46)		2 (40)	111 (46)		3 (60)	111 (46)	
≥1 for males & ≥1.3 for females	43 (43)	132 (54)		118 (43)	132 (54)		3 (60)	132 (54)		2 (40)	132 (54)	
Triglycerides, n (%)			0.121			0.791			0.801			0.801
<1.7	44 (44)	132 (54)		144 (53)	132 (54)		3 (60)	132 (54)		3 (60)	132 (54)	
≥1.7	55 (56)	111 (46)		128 (47)	111 (46)		2 (40)	111 (46)		2 (40)	111 (46)	
High cholesterol	65 (66)	98 (40)	<**0**.**001**	136 (50)	98 (40)	**0**.**033**	4 (80)	98 (40)	0.162	3 (60)	98 (40)	0.400
Hypertension	31 (31)	97(40)	0.142	126 (46)	99 (41)	0.213	1 (20)	99 (41)	0.651	2 (40)	99 (41)	0.073
Systolic blood pressure, mean ± SD	127 ± 18	130 ± 21	0.154	134 ± 22	130 ± 21	**0**.**025**	113 ± 12	130 ± 21	0.070	125 ± 17	130 ± 21	0.605
Diastolic blood pressure, mean ± SD	84 ± 10	83 ± 12	0.701	87 ± 13	83 ± 12	**0**.**003**	78 ± 12	83 ± 12	0.258	82 ± 13	83 ± 12	0.744

After comparing UAE national diabetics versus diabetics from other ethnicities, we carried a univariate analysis to compare diabetic and non-diabetic subjects within each ethnicity (Table 2). From this univariate analysis, age and gender variables together with any other variables that in univariate analysis were significant at *P* < 0.05 were selected for multivariate regression analysis: ethnicity, family history of diabetes, physical activity, snoring, obesity, triglyceride levels, high density lipoprotein levels, cholesterol levels, systolic and diastolic blood pressure.

**Table 2:** Univariate factors of diabetes versus non-diabetes across different ethnicities of UAE national patients and immigrant ethnic groups of Arab non-nationals, Asian non-Arabs, Westerners and African.

Variable	Arab non-national			Asian			Westerners			Africans			UAE nationals		
	DM n = 99	No DM n = 541	*P* value	DM n = 272	No DM n = 1411	*P* value	DM n = 5	No DM n = 51	*P* value	DM n = 5	No DM n = 22	*P* value	DM n = 243	No DM n = 554	*P* value
Age (years) mean ± SD	49 ± 10	37 ± 11	<**0**.**001**	44 ± 11	36 ± 9	<**0**.**001**	39 ± 16	42 ± 13	0.649	38 ± 13	35 ± 9	0.546	51 ± 14	39 ± 12	<0.001
Male, n (%)	67 (68)	392 (72)	0.331	237 (87)	1201 (85)	0.338	2 (40)	32 (63)	0.320	3 (60)	13 (59)	0.970	131 (54)	288 (52)	0.617
Female, n (%)	32 (32)	149 (28)	0.331	35 (13)	210 (15)	0.338	3 (60)	19 (37)	0.320	2 (40)	9 (41)	0.970	112 (46)	266 (48)	0.617
First-degree family history of diabetes, n (%)	55 (56)	203 (38)	**0**.**001**	91 (33)	241(17)	<**0**.**001**	0	12 (24)	0.221	3 (60)	5 (23)	**0**.**099**	155 (64)	319 (58)	0.100
Activity, n (%)	62 (63)	317 (59)	0.453	136 (50)	578 (41)	**0**.**006**	2 (40)	29 (57)	0.469	3 (60)	9 (41)	0.438	55 (23)	172 (31)	**0**.**015**
Snoring, n (%)	39 (39)	134 (25)	**0**.**003**	78 (29)	173 (12)	<**0**.**001**	2 (40)	11 (22)	0.352	0	1 (5)	0.627	231 (95)	541 (98)	0.053
BMI (kg/m^2^), n (%)			**0**.**003**			<**0**.**001**			0.380			0.155			<**0**.**001**
Normal	13 (13)	117 (22)		87 (32)	629 (45)		1 (20)	19 (37)		2 (40)	9 (41)		43 (18)	158 (29)	
Overweight	31 (31)	219 (40)		117 (43)	596 (42)		2 (40)	24 (47)		0	8 (36)		80 (33)	212 (38)	
Obese	55 (56)	204 (38)		66 (24)	182 (13)		2 (40)	8 (16)		3 (60)	5 (23)		118 (49)	182 (33)	
Waist circumference high, n (%)	64 (65)	237 (44)	<**0**.**001**	200 (74)	849 (60)	<**0**.**001**	2 (40)	12 (24)	0.417	3 (60)	9 (41)	0.548	152 (63)	253 (46)	<**0**.**001**
High cholesterol	65 (66)	281 (52)	**0**.**012**	136 (50)	711 (50)	0.906	4 (80)	24 (47)	0.160	3 (60)	8 (36)	0.332	98 (40)	246 (44)	0.285
HDL, n (%)			0.106			**0**.**014**			0.079			0.726			<**0**.**001**
<1 for males & <1.3 for females	43 (43)	189 (35)		118 (43)	501 (36)		3 (60)	12 (24)		2 (40)	7 (32)		132 (54)	206 (37)	
≥1 for males & ≥1.3 for females	56 (57)	352 (65)		154 (57)	910 (64)		2 (40)	39 (76)		3 (60)	15 (68)		111 (46)	348 (63) **	
Triglycerides, n (%)			<**0**.**001**			<**0**.**001**			0.352			0.171			<**0**.**001**
<1.7	44 (44)	362 (67)		144 (53)	912 (65)		3 (60)	40 (78)		3 (60)	19 (86)		132 (54)	432 (78)	
≥1.7	55 (56)	179 (33)		128 (47)	499 (35)		2 (40)	11 (22)		2 (40)	3 (14)		111 (46)	122 (22)	
Hypertension	31 (31)	110 (20)	**0**.**017**	126 (46)	477 (34)	<**0**.**001**	1 (20)	14 (27)	0.720	2 (40)	5 (23)	0.426	99 (41)	103 (19)	<**0**.**001**
Systolic blood pressure, mean ± SD	127 ± 18	120 ± 16	<**0**.**001**	134 ± 22	127 ± 17	<**0**.**001**	113 ± 12	121 ± 21	0.463	125 ± 17	123 ± 18	0.751	130 ± 21	123 ± 55	<**0**.**001**
Diastolic blood pressure, mean ± SD	84 ± 10	80 ± 11	<**0**.**001**	87 ± 13	84 ± 12	<**0**.**001**	78 ± 12	81 ± 16	0.660	82 ± 13	79 ± 11	0.676	83 ± 12	84 ± 68	<**0**.**001**

BMI, body mass index; DM, diabetes mellitus; HDL, high density lipoprotein; SD, standard deviation; UAE, United Arab Emirates.

In multivariate analysis, older age was associated with increased odd ratio (OR) of diabetes with odds increasing from 2.70 (95% confidence interval [CI] 1.96–3.72) for 41–50 years age group to 6.08 (95% CI 4.28–8.64) for 51–60 years age group, and reaching OR of 8.26 (95% CI 5.32–12.82) for age group of 60 years and older. Table 3 shows the adjusted values for logistic regression analysis.

**Table 3:** Multivariate factors of diabetes versus non-diabetes.

	Adjusted OR (95% CI)	P Value
Male	0.86 (0.67–1.12)	0.864
**Age**		
18–30	Reference	Reference
31–40	1.01 (0.73–1.40)	0.951
41–50	2.70 (1.96–3.72)	<0.001
51–60	6.08 (4.28–8.64)	<0.001
60+	8.26 (5.32–12.82)	<0.001
**Ethnicity**		
Arab non-national	Reference	Reference
Asian	1.62 (1.21–2.17)	0.001
African and westerner	1.02 (0.48–2.17)	0.952
Arab national	1.57 (1.10–2.22)	0.012
Family history of diabetes	1.84 (1.48–2.27)	<0.001
Physical activity	1.09 (0. 89–1.34)	0.403
Snoring	1.40 (1.07–1.84)	0.015
Obese	1.60 (1.28–1.99)	<0.001
High cholesterol	0.86 (0.70–1.05)	0.145
Triglycerides (mmol/L)	1.25 (1.15–1.35)	<0.001
HDL (mmol/L)	0.64 (0.45–0.91)	0.013
Systolic blood pressure, per increase of 10 mmHg	1.02 (1.01–1.02)	0.004
Diastolic blood pressure, per increase of 10 mmHg	0.99 (0.98–0.99)	0.012

CI, confidence interval; HDL, high density lipoprotein; OR, odds ratio.

When comparing the OR for diabetes across the local and immigrant ethnicities, Asian non-Arabs (OR 1.62; 95% CI 1.21–2.17) and local UAE nationals (OR 1.57; 95% CI 1.10–2.22) were more likely associated with diabetes condition. In addition, family history of diabetes (OR 1.84; 95% CI 1.48–2.27), snoring (OR 1.40; 95% CI 1.07–1.84), obesity (OR 1.60; 95% CI 1.28–1.99), elevated levels of triglycerides (OR 1.25; 95% CI 1.15–1.35) as well as elevated systolic blood pressure (OR 1.02; 95% CI 1.01–1.02) was associated with increased odds of diabetes. In contrast, increased high density lipoprotein (OR 0.64; 95% CI 0.45–0.91) decreased the odds for diabetes whereas increased diastolic blood pressure (OR 0.99; 95% CI 0.98–0.99) appeared to decrease odds for diabetes but the value was close to non-significant.

## Discussion

The finding of the current study displays the heterogeneity presents among prevalence and characteristics for patients with diabetes coming from different ethnic backgrounds.

In this study the variation in age standardised prevalence of prediabetes and diabetes was shown across different ethnicities of local UAE nationals and immigrant non-nationals. The UAE’s immigrant population was mainly made up of Arab non-national and Asian non-Arabs with ethnic groups of westerners and Africans as minorities. Prediabetes ranged from around 8% to 17%, while the prevalence of newly diagnosed diabetes ranged from around 3% to 13%. UAE nationals, Arabs non-nationals and Asians had the highest number of prediabetes as well as newly diagnosed diabetes. Age standardised prevalence of diabetes was highest in national UAE locals as well as Asian non-Arabs, where 40% of both groups fell under the range of either prediabetes or diabetes conditions. Likewise, the total age standardised prevalence of diabetes was highest among Asians (male 23% and female 20%) and UAE locals (male 21% and female 23%). This prevalence was higher than the UAE diabetes prevalence of 17.3% that was reported in 2017 via IDF for the whole UAE population regardless of ethnicities. Moreover, compared to other Gulf countries, the prevalence in this study was higher than 18.3% prevalence reported by Bahijri *et al*. from Saudi Arabia^17^, the 18.8% prevalence reported by Alkandari *et al*. from Kuwait^18^, and diabetes prevalence from Oman that ranged from 10.5% to 17.7%^19^.

Another ethnic group with high diabetes prevalence was the Asians which was consistent to high prevalence reported via previous investigations^20^. Compared to UAE national’s diabetics, Asians tended to have lower BMI but larger waist circumferences which could result in more visceral fat, more insulin resistance and consequently more diabetes. This finding could also explain high prevalence of diabetes among Asians despite their lower BMI, which is keeping with literature that have reported lower BMI and more visceral fat in Asians with diabetes compared to western population^21^.

In the logistic regression analysis, diabetes was again associated mainly with two ethnicities of Arab national and Asian non-Arab. Other multivariate factors of diabetes were consistent with previous literature and included older age^22^, family history of diabetes^23,24^, obesity^25,26^, snoring^27^ higher TG and finally raised blood pressure^28^.

According to IDF the UAE diabetes prevalence is growing faster than the rest of MENA regions and expected to double in number by 2040^2^. It is recommended that health authorities in MENA regions to continue funding the research about ethnic specific diabetes characteristics and to increase the diabetes knowledge among healthcare and public sectors. The major driving factors of fast increase in diabetes prevalence among UAE local and immigrant ethnicities are the increased obesity, shift to sedentary lifestyle, decrease in physical activity and unhealthy diet^29–31^. There is an urgent need for increasing the knowledge and awareness of public as well as health care sector about diabetes, its risk factors and complications. The public should be encouraged to shift to healthier lifestyle, eat healthy diet that is low in sugar but high in fiber, and increase in physical and sport activities. It is important to understand that how diabetes prevalence and risk factors could vary across the different ethnic groups in UAE. Therefore, the interventions towards identification and prevention of diabetes development as well as progression should not treat all ethnicities alike. The data from this study can be used in conjunction with genetics to characterize genetic factors that might lead to prevalence to diabetes in various ethnic populations.

In summary, the prevalence and risk factors of diabetes vary across different ethnic groups in UAE. Local UAE nationals and Asians non-Arab ethnic groups have the highest prevalence of diabetes; more than one-third of these ethnic groups have either prediabetes or diabetes. Asians with diabetes have lower BMI but higher waist circumference. In addition, compared to local UAE nationals, non-nationals immigrants with diabetes tend to be younger, predominantly male and more active.
